# Anti-Lung Cancer Activity through Enhancement of Immunomodulation and Induction of Cell Apoptosis of Total Triterpenes Extracted from *Ganoderma luncidum* (Leyss. ex Fr.) Karst.

**DOI:** 10.3390/molecules18089966

**Published:** 2013-08-19

**Authors:** Liang Feng, Ling Yuan, Meng Du, Yan Chen, Ming-Hua Zhang, Jun-Fei Gu, Jun-Jie He, Ying Wang, Wei Cao

**Affiliations:** Key Laboratory of New Drug Delivery Systems of Chinese Materia Medica, Jiangsu Provincial Academy of Chinese Medicine, Nanjing 210028, Jiangsu, China; E-Mails: wenmoxiushi@163.com (L.F.); yuanl.china@sina.com (L.Y.); dumengwohaoaini@163.com (M.D.); jdyx0701.111@163.com (M.-H.Z.); gujunfei0123@126.com (J.-F.G.); a518518100@126.com (J.-J.H.); wangying9021@163.com (Y.W.); cwyxz_love@126.com (W.C.)

**Keywords:** *Ganoderma luncidum* (Leyss. ex Fr.) Karst., triterpenes, anti-lung cancer, immune function, apoptosis inducing

## Abstract

*Ganoderma luncidum* (Leyss. ex Fr.) Karst. (GLK) has been used traditionally for the prevention and treatment of cancers or tumors for a long time in Traditional Chinese Medicine. The triterpenes as main effective components of GLK have been found to be beneficial for the efficacy. The purpose of this study was to examine the anti-lung cancer activity of triterpenes of GLK *in vitro* and *in vivo* and to explore their anti-lung cancer effects and potential mechanisms. A549 cells and Lewis tumor-bearing mice were used to evaluate the inhibition effects of triterpenes on cell proliferation and tumor growth. The IC_50_ of triterpenes of GLK on A549 cells was 24.63 μg/mL. Triterpenes of GLK could significantly inhibit tumor growth in mice (30, 60 and 120 mg/kg). The immune organs indexes including spleen and thymus were increased remarkedly by the treatment with triterpenes. Moreover, they were able to stimulate the immune response by increasing the expressions of IL-6 and TNF-α. Flow cytometric analysis revealed that cell arrest caused by triterpenes treatment (7.5, 15 and 30 μg/mL) was in the G2/M phase in A549 cells. Triterpenes induced apoptosis by decreasing the expression of the antiapoptotic protein Bcl-2 and pro-caspase 9 and increasing the levels of cleaved-caspase 9. Our findings suggested that the triterpenes of GLK have anti-lung cancer activity* in vitro* and *in vivo* via enhancement of immunomodulation and induction of cell apoptosis. The study provides insights into the mechanism of GLK in the prevention and treatment of lung cancer.

## 1. Introduction

Lung cancer is one of the most frequently diagnosed cancers and the leading cause of cancer death worldwide [1]. Unfortunately, some current treatments involving non-selective cytotoxic chemotherapy, radiotherapy and surgery may result in only a modest increase in survival and a significant toxicity for the patient with lung cancer [2]. As known to all, Traditional Chinese Medicine has been found to be beneficial for the prevention and treatment of cancers/tumors through mutiple mechanisms, including immunostimulation and the induction of apoptosis [3]. The combined effects of the multi-component medicines may contribute to the inhibition of tumor growth. Additionally, they can also enhance the body’s immunity with low side effects and toxicity [4,5].

*Ganoderma luncidum* (Leyss. ex Fr.) Karst. (GLK) has been recorded in the old Chinese medical text *Shen Nong’s Herbal Classic* due to its medicinal value [6,7]. It has been used traditionally for treating fatigue, cough, asthma, insomnia, indigestion, *etc*. [8]. Recently, its anti-tumor activity has attracted the attention of many researchers [9,10,11]. Phytochemical studies show it contains bioactive triterpenes, including ganoderic acid B, ganoderenic acid A, ganoderic acid A, lucideric acid A, *etc*. [12,13,14,15,16]. Accumulating evidence has shown that the triterpenes of GLK can inhibit the proliferation of hepatoma cells and HeLa cells [17,18], as well as human colon cancer cells HT-29 [19]. However, the potential anti-lung cancer activity of triterpenes of GLK and its possible mechanism remain unclear.

Therefore, the aim of this study was to evaluate the anti-lung cancer effect of these triterpenes *in vivo* and *in vitro*, and also explore the underlying mechanisms.

## 2. Results

### 2.1. Identification of Triterpenes by HPLC-DAD and LC/MS Analysis

**Figure 1:**
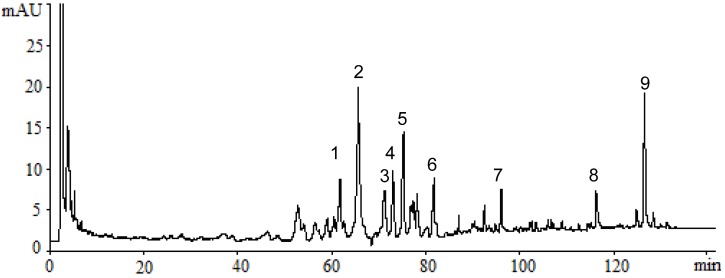
HPLC chromatograms of triterpenes in extract of GLK. (1) ganoderic acid C2; (2) ganoderic acid G; (3) Ganoderic acid C6; (4) ganoderic acid B; (5) ganoderenic acid A; (6) ganoderic acid A; (7) lucideric acid A; (8) ganoderenic acid D and (9) ganoderic acid C1.

**Figure 2:**
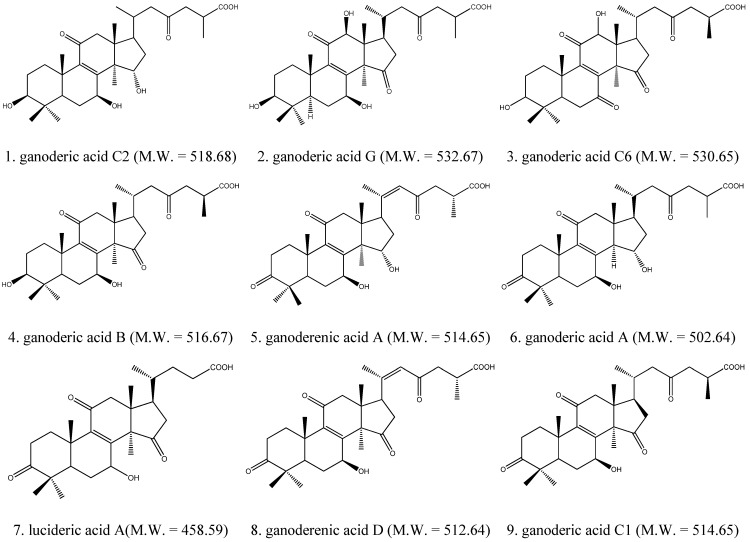
The chemical structures of triterpenes of GLK.

**Figure 3:**
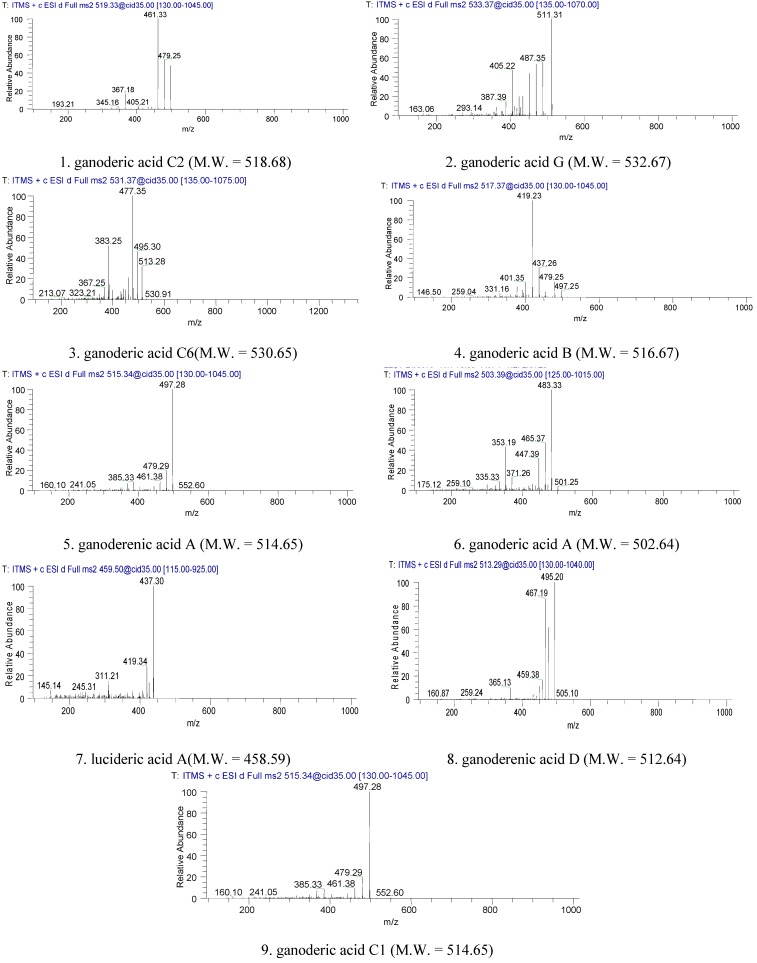
The MS^2^ of LC/MS ion fragmentation of triterpenes of GLK.

The HPLC-DAD chromatogram of a supercritical CO_2_ fluid extract of the fruiting bodies of GLK is shown in Figure 1. The chromatogram showed that triterpenes of GLK could be eluted completely under the HPLC conditions used within 128 min, and separated satisfactorily. The peaks whose retention time ranges from 50 min to 128 min might consist mainly of triterpenes. LC/MS analysis was performed to identify these compounds, which were identified as ganoderic acid C2; ganoderic acid G; Ganoderic acid C6; ganoderic acid B; ganoderenic acid A; ganoderic acid A; lucideric acid A; ganoderenic acid D and ganoderic acid C1 (Figure 2 and Figure 3).

### 2.2. Inhibitory Effect of Triterpenes on A549 Cell Proliferation

**Figure 4:**
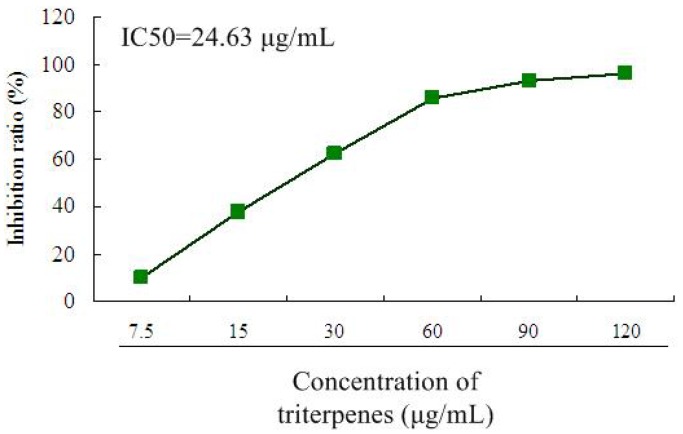
Proliferation inhibition effect of triterpenes on A549 cells after treatment for 36 h. The IC_50_ of triterpenes on A549 cells was calculated by the SPSS 11.5 software. All data were expressed as means ± SD (*n* = 12).

The MTT method was used for the evaluation of proliferation inhibition activity *in vitro* [20]. As can be seen in Figure 4, the treatment with triterpenes (7.5, 15, 30, 60, 90, 120 μg/mL) showed significant proliferation inhibition effects on A549 cells in a dose-dependent manner after treatment for 36 h. The IC_50_ of triterpenes on A549 cells was 24.63 μg/mL. The findings of the MTT assay showed that the triterpenes exhibited high anti-proliferative effects against A549 cells, indicating that the triterpenes of GLK could contribute to the reduction of the cell viability of A549 cells.

### 2.3. Anti-Tumor Activity of Triterpenes in Lewis Tumor-Bearing Mice

**Figure 5:**
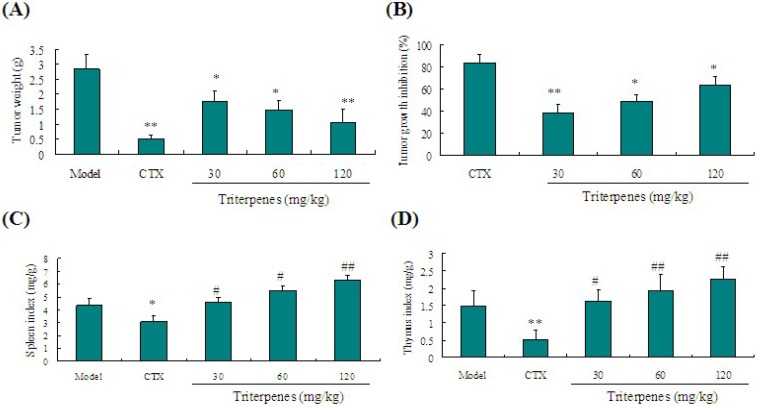
Effect of triterpenes of GLK on tumor growth and immune organ indexes in Lewis tumor-bearing mice. (**A**) tumor weight; (**B**) inhibition ratio of tumor growth; (**C**) spleen index; (**D**) thymus index. All data were taken from different individual experiments and expressed as means ± SD (*n* = 8).

Lewis tumor-bearing C57BL/6 mice were used to verify the anti-lung cancer activities of triterpenes *in vivo*. As shown in Figure 5A, a significant decrease of tumor weight in the triterpenes-treated group was observed compared to the model mice (*p* < 0.05). Triterpenes of GLK (30, 60 and 120 mg/kg) significantly inhibited tumor weights in mice by 38.03%, 48.24% and 63.38% compared to the model mice (Figure 5B). Our findings suggested that the triterpenes of GLK held a significant tumor inhibition effect on tumor-bearing mice.

### 2.4. Immunostimulation Activity of Triterpenes on Spleen and Thymus Indexes in Lewis Tumor-Bearing Mice

Immunostimulation has been considered as one of potential mechanisms leading to the tumor growth inhibition. Although traditional chemotherapy is beneficial against tumor growth, its side effects, including immunosuppressive effects and toxicity limit its use in clinic. As shown in Figure 5C,D, the positive control cyclophosphamide (CTX, 20 mg/kg) could inhibit the immunological function of mice through decreasing the indexes of spleen and thymus. However, these decreases were reversed to a normal level by treatment with triterpenes of GLK (30, 60 and 120 mg/kg) (*p* < 0.05). These results indicated that the triterpenes of GLK might at least partially inhibit tumor growth via stimulation of the immune response.

### 2.5. Regulation of Triterpenes on IL-6 and TNF-α Levels in Lewis Tumor-Bearing Mice

The potential immunomodulatory activities of the triterpenes were determined by assessing the cytokine-production profiles by ELISA. As shown in Table 1, compared with those of the >CTX and >model groups, the IL-6 and TNF-α levels of mice treated with triterpenes (30, 60 and 120 mg/kg) were significant higher. This suggested that the immunomodulation activities of triterpenes might be associated with the upregulation of the expression levels of IL-6 and TNF-α.

**Table 1:** Comparison of TNF-α and IL-6 of triterpenes *in vivo*.

Groups	Concentration of TNF-α (pg/mL)	Concentration of IL-6 (pg/mL)
Model	42.07 ± 0.86 ^#^	34.53 ± 7.89 ^#^
CTX (20 mg/kg)	31.72 ± 7.87 ^*^	20.60 ± 1.60 ^*^
Triterpenes (30 mg/kg)	46.84 ± 4.30 ^#^	38.50 ± 7.86 ^#^
Triterpenes (60 mg/kg)	50.44 ± 3.2 ^*,#^	42.78 ± 5.64 ^*,#^
Triterpenes (120 mg/kg)	56.77 ± 6.32 ^*,#^	48.59 ± 3.45 ^*,#^

^*^ p < 0.05 *vs*. model group; ^#^* p* < 0.05 *vs.* CTX group. All data were expressed as means ± SD, *n* = 8.

### 2.6. Induction of Triterpenes on Apoptosis of A549 Cells

**Figure 6:**
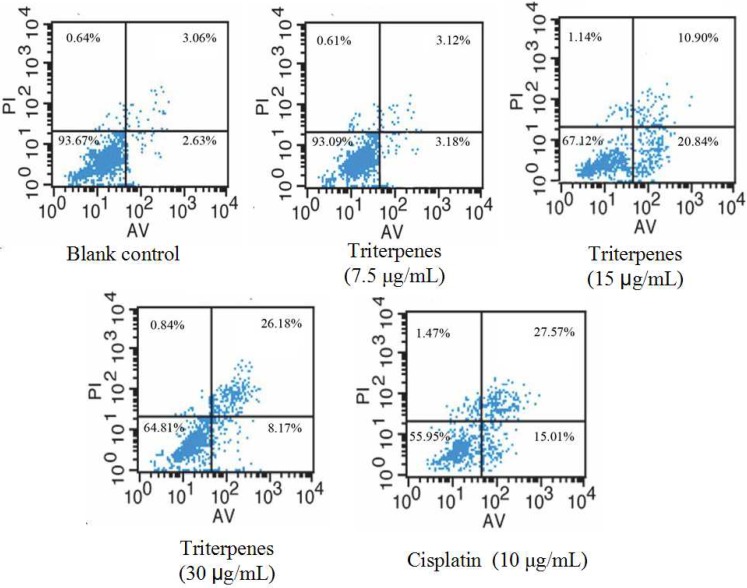
Induction of triterpenes of GLK on A549 cells apoptosis. Cells were treated with triterpenes of GLK at a concentration of 7.5 μg/mL, 15 μg/mL and 30 μg/mL for 36 h and then co-stained with PI and FITC-conjugated annexin V.

The Annexin V-FITC apoptosis detection kit was used to examine by flow cytometry the induction of A549 cell apoptosis by GLK triterpenes. Annexin V/PI by flow cytometry contained four quadrants. The Q3 quadrant represents normal cells because the cells in this quadrant are viable and negative for both PI/annexin V. Q4 represents early apoptotic cells which are positive for annexin V and negative for PI, while Q2 represents late apoptotic cells which are positive for annexin V and PI. The upper right (Q2) quadrant represents late apoptotic cells while the lower right quadrant (Q4) represents early ones. The total apoptosis rate was the sum of late apoptosis in Q2 and early apoptosis in Q4. As depicted in Figure 6, the percentage of total apoptosis in A549 cells was remarkably increased by the treatment with triterpenes (7.5 μg/mL for 6.30%; 15 μg/mL for 31.74%; 30 μg/mL for 34.35%). The apoptotic percentage of A549 cells by 15 μg/mL concentration was mostly in early stage while they were mostly distributed in the late stage by 30 μg/mL concentration. The late apoptotic ratio was enhanced significantly in a concentration-dependent manner. For early apoptosis, the apoptotic ratio was increased at a concentration of 15 μg/mL. However, there is a decrease at 30 μg/mL concentration. This might be related to the transformation of early apoptosis to late apoptosis. Taken together, these findings indicated that the triterpenes of GLK could induce A549 cell apoptosis to inhibit tumor growth in A549 cells.

### 2.7. Effect of Triterpenes on Cell Cycle Arrest of A549 Cells

**Figure 7:**
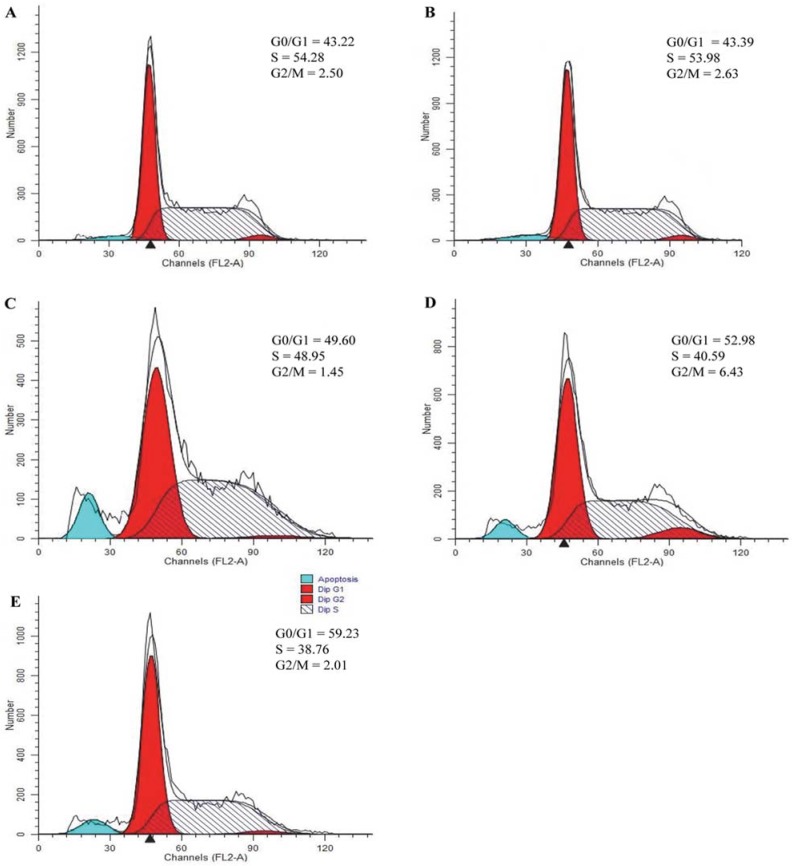
The effect of triterpenes on cell cycle of A549 cells. Cells were treated with blank control (**A**); 7.5 μg/mL triterpenes (**B**); 15 μg/mL triterpenes (**C**); 30 μg/mL triterpenes (**D**); positive control CTX (10 μg/mL) (**E**) for 36 h, and then cell cycle analysis was determined by flow cytometry.

To determine whether the induction apoptosis of triterpenes was related to the arrest of cell cycle progression in A549 cells, flow cytometry was used to quantitate the cell cycle distribution under treatment with the triterpenes at the concentration of 7.5, 15 and 30 μg/mL for 36 h. As depicted in Figure 7, the treatment with triterpenes could induce apoptosis on A549 cells via G1/S cell cycle arrest. The triterpenes decreased the cells in S phase, whereas it increased the cells in the G1 and G2 phases. The results showed the triterpenes could regulate the cell cycle to induce cell apoptosis.

### 2.8. Apoptosis-Related Protein Expressions

**Figure 8:**
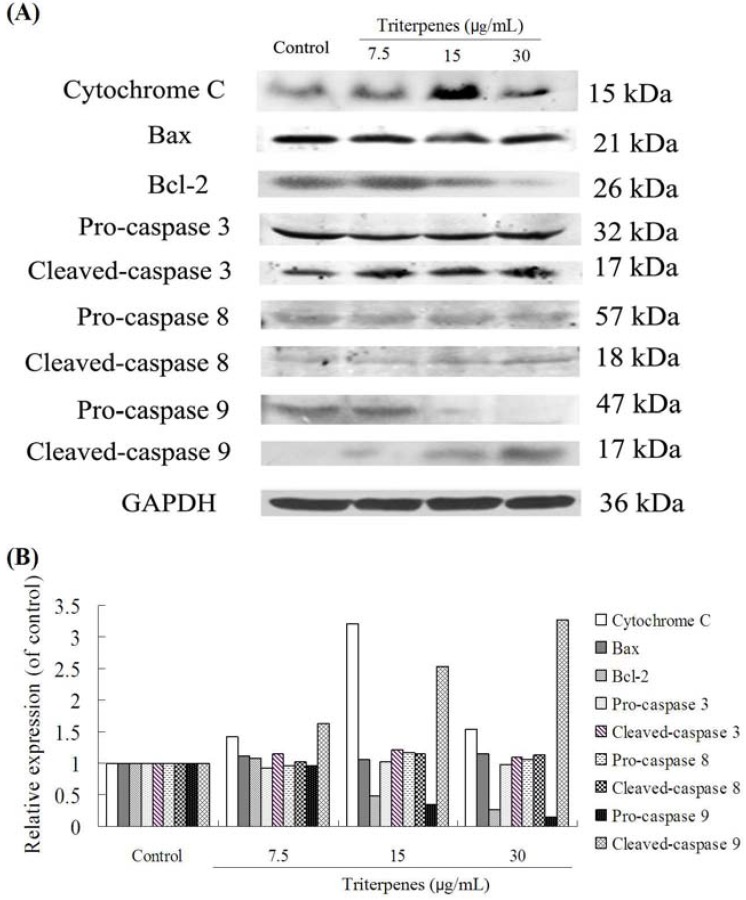
Expressions of apoptosis-related proteins after treating with triterpenes in A549 cells. (**A**) brands of western blotting; (**B**) the relative expressions.

The possible signaling mechanisms involved in the extract-induced apoptosis of A549 cancer cells were determined by western blotting analysis of standard apoptosis-related proteins, including pro-caspase 3, cleaved-caspase 3, pro-caspase 8, cleaved-caspase 8, pro-caspase 9, cleaved-caspase 9, Bcl-2, Bax, and cytochrome C. As shown in Figure 8A,B, the triterpenes of GLK (7.5, 15 and 30 μg/mL) decreased significantly the expression of the antiapoptotic protein Bcl-2 and pro-caspase 9 in a concentration-dependent manner. Furthermore, the triterpenes treatment increased the expression levels of cleaved-caspase 9. For cytochrome C expression, triterpenes at 15 μg/mL increased significantly its expression, whereas 30 μg/mL concentration decreased it. This might be associated with the effect of concentration on the target and its signal transduction events. In our experiments, the GLK triterpenes could affect the expressions of some proteins, including Bax, pro-caspase 3, cleaved-caspase 3, pro-caspase 8 and cleaved-caspase 8. Our findings indicated that the induction of triterpenes on apoptosis might be associated with Bcl-2, pro-caspase 9 and cleaved-caspase 9.

## 3. Discussion

It is well known that chemotherapy or radiotherapy causes immunosuppression in patients with cancer [21]. However, the advantage of Traditional Chinese Medicine in cancer treatment is not only the inhibition of tumor growth, but also by causing immunostimulation for antitumor activity [4]. Our study found the the triterpenes of GLK could increase the immunity indexes, including the thymus and spleen indexes. The improvement of immune organs indexes was thought to be exerted via immunostimulation [22]. Moreover, cytokine productions (IL-6 and TNF-α) are responsible for enhancing antitumor immunity [23]. The results suggested that the antitumor activity of triterpenes of GLK was probably related to its regulation on the levels of IL-6 and TNF-α.

Apoptosis is recognized as an efficient strategy for tumor chemotherapy and has been considered as an indicator for the prevention and treatment of tumors [24]. Flow cytometric analysis showed the triterpenes of GLK induced apoptosis on A549 cells via G1/S cell cycle arrest. The potent anti-lung cancer activity of the triterpenes of GLK might be attributed to this apoptosis mechanism. Generally, cell cycle arrest and apoptosis are connected, that is, occurrence of cell cycle arrest leads to cell apoptosis, which involves numerous signaling molecules and regulatory proteins. Bcl-2 is an upstream effector molecule in the mitochondria-mediated intrinsic apoptotic mechanism and is defined as a potent suppressor of apotosis [25]. We found that the triterpene treatment downregulated the protein level of antiapoptotic protein Bcl-2 and pro-caspase 9 in A549 cells, whereas it upregulated the levels of cleaved-caspase 9. This observation provided insights into the underlying molecular mechanisms of the triterpenes of GLK on its anticancer activity.

We showed that the triterpenes of GLK inhibited cell growth and that this was correlated with increased G1/S cell cycle arrest. It had been confirmed that GLK triterpenes both concentration- and time-dependently mediate G1 cell cycle arrest and significantly decrease the protein level of CDK2, CDK6, cycle D1, p-Rb and c-Myc in MCF-7 human breast cancer cells [26]. The results of Li *et al*. demonstrated that ganoderic acids of GLK suppressed growth and angiogenesis in breast cancer cells by modulating the NF-κB signaling pathway including c-Myc and cyclin D1, anti-apoptosis Bcl-2, MMP-9 and VEGF [27]. In addition, GLK triterpenes can also stimulate caspase-3 and caspase-9 activity and up-regulate Bax/Bcl-2 ratio in HeLa cells [28]. Recently, Yao *et al*. revealed that the triterpenes of GLK might also enhance chemosensitivity of HepG2 cells to cisplatin by inhibiting the JAK-STAT3 signaling pathway [14]. Ganoderic acid from GLK mycelia has been shown to induce mitochondria mediated apoptosis in lung cancer cells [29]. Combined with the potential mechanism of GLK triterpenes on other tumor cells, we conclude that GLK can induce apoptosis of lung cancer cells through the JAK-STAT3-NF-κB signaling pathway. This may be beneficial for the induction of apoptosis of lung cancer cells by GLK.

## 4. Experimental

### 4.1. Chemicals and Reagents

Fetal bovine serum (FBS), RPMI 1640 medium and trypsin were purchased from Gibco/BRL (Grand Island, NY, USA). 3-(4,5-Dimethylthiazol-2-yl)-2,5-diphenyltetrazolium bromide (MTT) and dimethyl sulfoxide (DMSO) were purchased from Sigma Chemical (St. Louis, MO, USA). Cisplatin and cyclophosphamide were obtained from Jiangsu Hengrui Medicine Co., Ltd. (Lianyungang, China). HPLC grade methanol, acetonitrile and phosphoric acid (TEDIA, USA) were used as mobile phase. Remaining reagents were AR grade. Antibodies to caspase-3, caspase-8, caspase-9, Bcl-2, Bax, cytochrome C, and glyceraldehyde-3-phosphate dehydrogenase (GAPDH) were purchased from Cell Signaling Technology (Beverly, MA, USA). Horseradish peroxidase (HRP)-conjugated secondary antibodies were obtained from Santa Cruz Biotechnology (San Diego, CA, USA).

### 4.2. Plant Materials

The dried fruiting bodies of *Ganoderma luncidum* (Leyss. ex Fr.) Karst. were purchased from Yanchen Shennong Healths Foodstuffs Co., Ltd. (Jiangsu, China) and authenticated by Professor Q. N. Wu, from the Nanjing University of Chinese Medicine. The specimens were stored in our laboratory under standard conditions.

### 4.3. Preparation of Plant Extracts

Total triterpenes in the fruiting bodies of GLK was extracted by HA221-40(50)-25 supercritical CO_2_ fluid (Nantong Hua’an SFE Limited Company, Jiangsu, China). The optimal conditions for extraction were as follows: pressure: 30 MPa, temperature: 45 °C, CO_2_ flow rate: 23 L/h, dynamic extraction time: 1 h.

### 4.4. Chromatographic Analysis of Triterpenes

A HPLC-DAD method was used to analyze the total triterpenes in GLK extract. HPLC-DAD analysis was performed on an Agilent 1100 series system (Agilent, Wilmington, DE, USA), equipped with a diode array detector (DAD), a quaternary pump and an automatic sample injector. The analysis was performed on a ZORBAX SB-C_18_ column (4.6 × 250 mm, 5 μm). The column temperature was set to 30 °C. Acetonitrile (A) and 0.05% phosphoric acid (B) were used for the mobile phase. The elution gradient for the total triterpenes was as follows: 0–40 min, 27% A; 40–60 min, 27%–35% A; 60–80 min, 35%–45% A; 80–90 min, 45%–65% A; 90–100 min, 65%–75% A; 100–110 min, 75%–85% A; 110–120 min, 85%–100% A; 120–128 min, 100% A. The flow rate was set at 1.0 mL/min. The detection wavelength was set at 254 nm and aliquots of 10 μL were injected. In addition, HPLC-ESI-MS was used to analyze the triterpenes in the extract of GLK. LC/MS analysis was performed on a Finnigan LCQ Fleet ion trap mass spectrometer with Xcal-ibur version 2.0 controlling software (Thermo Finnigan, San Jose, CA, USA), equipped with an electrospray ionization (ESI) interface. Analysis was carried out under positive ion mode. The detailed analysis parameters are set as follows: High purity N_2_ was used as the nebulizing gas while ultra-high purity He as the collision gas. Ion spray voltage was kept at −4.5 kV and capillary temperature at 300 °C. The flow rate of sheath gas (N_2_) was set at 40 a.u. while auxiliary gas (N_2_) at 10 a.u. Additionally, capillary voltage was −22 V and tube lens offset voltage was −60 V. The full-scan MS data were recorded within the range of m/z from 100 to 1,000.

### 4.5. Cell Culture

Human lung adenocarcinoma cell line A549 and mice Lewis lung carcinoma cells were obtained from KeyGEN Biotech (Nanjing, China). A549 cells were maintained in Dulbecco’s modified Eagle’s medium (DMEM) supplemented with 10% fetal calf serum (FCS), 1% nonessential amino acids, 100 U/mL penicillin, and 100 μg/mL streptomycin in a humidified atmosphere of 5% CO_2_ at 37 °C. Lewis cells were incuabetd in RPMI-1640 medium supplemented with 10% FCS, 100 U/mL penicillin, and 100 μg/mL streptomycin. Lewis cells generated a 80%–90% confluent layer and were prepared to suspension for animal experiment.

### 4.6. Cell Proliferation Assay

Cell viability was determined using the 3-(4,5-dimethylthiazol-2-yl)-2,5-diphenyltetrazolium bromide (MTT) assay. Human lung adenocarcinoma A549 cells (5 × 10^3^ cells/well) were seeded in 96-well plates. After 24 h, the cells were treated with triterpenes at different concentrations. After treatment for 36 h, 10 μL of MTT (5 mg/mL) was added to each well and incubated for other 4 h at 37 °C. The violet crystals were solubilized in DMSO (100 μL/well). After being shaken in a shaker, the absorbance of samples was measured on a microplate reader (Thermo Labsystems, Helsinki, Finland) at 550 nm. 50% inhibition concentration (IC_50_) of triterpenes was calculated by plotting the percentage of cell survival. In addition, the morphological changes of treated cancer cells were observed under COIC XDS-1B inverted phase contrast microscope (Chongqing Guang Dian Equipment Co., Ltd., Chongqing, China).

### 4.7. Anti-Tumor Activity on Lewis Tumor-Bearing C57BL/6

Male C57BL/6 mice (18–22 g) were obtained from the SLAC Lab Animal Center (Shanghai, China). The animal experiment protocol was reviewed and approved by the Institutional Animal Care and Use Committee of the Jiangsu Provincial Academy of Chinese Medicine. Lewis cells (1 × 10^7^ cells/mL) were injected subcutaneously in right armpit with 0.2 mL. The next day, the mice were randomly divided into groups and administered for 14 consecutive days: model group; positive control cyclophosphamide (20 mg/kg) group; triterpenes (30, 60 and 120 mg/kg). Model mice were treated orally with saline solution (0.9%) each day. Fourteen days later, the blood samples were taken from retinal vein for the determination of TNF-α and IL-6 by ELISA kits (Nangjing KeyGEN Biotech. Co., Ltd., Nanjing, China). After being sacrificed, the tumor, thymus and spleen were separated and weighed immediately. The inhibition ratio were calculated using the following formula [24]:

Inhibitory ratio (%) = [(A − B)/A] × 100%

where A and B are the average tumor weights of the model and treated groups, respectively. The immune organic indexes were calculated as organ weight/body weight [25].

### 4.8. Flow Cytometric Analysis on Cell Cycle

To investigate effects of triterpenes on the cell cycle distribution, A549 cells (6 × 10^5^ cells/mL) were treated with triterpenes at different concentration and then cultured for 36 h, respectively. The treated cells were harvested by centrifugation, washed with ice-cold phosphate-buffer saline (PBS) and then fixed in 70% ethanol overnight. After being washed twice with cold PBS, cells were treated with RNase (10 μg/mL) at 37 °C, and then stained with 10 μg/mL propidium iodide (PI) for 30 min in the dark. The DNA content was measured by FACScan flow cytometry (Becton-Dickinson, Mountain View, CA, USA).

### 4.9. Measurement of Apoptotic Ratio of A549 Cells

The apoptotic effects of triterpenes of GLK on A549 cells were determined by the Annexin V-FITC staining method and flow cytometry. A549 cells (6 × 10^5^ cells/mL) were treated with triterpenes and cultured for 36 h. The treated cells were harvested using trypsin, washed with PBS thrice, and resuspended in 100 μL binding buffer. Annexin V-FITC/PI apoptosis detection kit (KeyGEN, Nanjing, China) was used for the detection of apoptosis. Five microliters of annexin-fluorescein isothiocyanate (FITC) and 5 µL of propidium iodide (PI) solution were added, and incubated in the dark at room temperature for 15 min. Cell apoptosis was analyzed using FACScan flow cytometry (Becton Dickinson).

### 4.10. Western Blot Analysis

Western blotting was performed to detect the proteins of caspase-9, -8, -3, cytochrome C and Bcl-family. A549 cells (2 × 10^6^ cells/well) were seeded in 6-well culture dishes and treated with the indicated concentrations of triterpenes for 36 h. Cells were harvested and washed twice with ice-cold PBS, and then lysed for 5 min at 4 °C with ice-cold RIPA buffer (1% NP-40 in 150 mM NaCl, 50 mM Tris, and 2 mM ethylenediaminetetraacetic acid [EDTA]). Equalized amounts of proteins from each sample were subjected to sodium-dodecyl sulfate (SDS)-polyacrylamide gel electrophoresis. Protein bands were then transferred to polyvinylidene difluoride (PVDF) membranes. Membranes were blocked with 1% (w/v) bovine serum albumin (BSA) for 2 h, washed in TBST (50 mM Tris, 150 mM NaCl, 0.05% Tween 20, pH = 7.4) thrice, and incubated with primary antibodies caspase-3 (1:1000), caspase-8 (1:1,000), caspase-9 (1:1,000), Bcl-2 (1:1,000), Bax (1:1,000), cytochrome C (1:1,000), and GAPDH (1:1,000) overnight at 4 °C. The membranes were washed and incubated with the secondary antibody conjugated with IgG-HRP for 1 h at room temperature and then washed in TBST thrice. Immune complexes were detected using an enhanced chemiluminescence system. GAPDH was used as the loading control. The relative expressions of apoptotic proteins was quantified by Image pro plus (IPP) software.

### 4.11. Statistical Analysis

All data were analyzed using SPSS 11.5 software (IBM, Armonk, NY, USA). One-way analysis of variance was used for multiple comparisons, and Student’s t-test was used to compare two groups. A value of *p* < 0.05 was considered statistically significant. All values were expressed as means ± standard deviation (SD).

## 5. Conclusions

Our investigation evaluated the anti-lung cancer activities of the triterpenes of GLK *in vivo* and *in vitro*. Taken together, our results demonstrated that the antitumor effects might be related to the enhancement of immunomodulation and induction of apoptosis through mediation of cell cycle arrest and apoptosis-related protein expressions. Our study sheds light on the anti-cancer mechanism of the triterpenes of GLK from a molecular perspective point of view and provides useful information for the possible use of GLK in the clinic for complementary therapy of lung cancer.
